# Study of the Power Generation Performance of Impact Piezoelectric Energy Capture Devices

**DOI:** 10.3390/mi14051013

**Published:** 2023-05-08

**Authors:** Xiaochao Tian, Jinlong Liu, Jun Hou, Houjun Gai, Jie Yang, Zhenwen Sun

**Affiliations:** School of Mechanical and Vehicle Engineering, Changchun University, Changchun 130022, China

**Keywords:** piezoelectric energy capture, piezoelectric film, dynamics analysis, stimulation force

## Abstract

In order to solve the problem of conventional energy shortages, a non-resonant impact piezoelectric energy capture device using a (polyvinylidene fluoride) piezoelectric film at low frequency is proposed, and related theoretical analysis and experimental studies are conducted. The device has a simple internal structure, is green and easy to miniaturize, and is capable of harvesting energy at low frequencies to supply energy to micro and small electronic devices. First, to verify the feasibility of the device, the structure of the experimental device is modeled and dynamically analyzed. Then the modal, stress–strain, and output voltage of the piezoelectric film are simulated and analyzed using COMSOL Multiphysics simulation software. Finally, the experimental prototype is built according to the model, and the experimental platform is constructed to test the relevant performance. The experimental results show that the output power produced by the capturer varies within a certain range when the capturer is excited externally. With an external excitation force of 30 N, a piezoelectric film bending amplitude of 60°, and a piezoelectric film size of 45 × 80 mm, the resulting output power voltage is 21.69 V, the output current is 0.07 mA, and the output power is 1.5176 mW. This experiment verifies the feasibility of the energy capturer and provides a new idea for powering electronic components.

## 1. Introduction

In recent years, with the advancement of technology, traditional energy sources are in negative growth, and at the same time, cause greater pollution to the environment. From the perspective of sustainable development, energy has become an important material basis for the development of today’s society and plays a vital role in economic development, livelihood improvement, and national security. Therefore, the research and development of new energy harvesting technologies has received a lot of attention around the world, and many domestic and foreign scholars have used piezoelectric materials for energy capture because of their green and pollution-free characteristics [1,2,3,4]. Currently, there are various ways of energy harvesting technology, such as electromagnetic energy capture, piezoelectric energy capture, electrostatic energy capture, etc., using the properties of materials to complete the production of energy capture devices. With the rapid development of the Internet of Things, piezoelectric materials are widely used in various fields because of their high sensitivity and high energy density. In the field of energy harvesting, the generated energy is often used to supply energy for tiny electronic devices and sensors. Therefore, piezoelectric materials can be used as captive energy devices to solve the existing problems of energy pollution and non-renewable energy consumption, and have good prospects for development in the future.

Shock vibration is a common phenomenon in life and can be combined with piezoelectric technology for energy capture, which has a very high utilization value. Since the piezoelectric material selected for the shock vibration process requires high mechanical strength and elasticity, PVDF piezoelectric film with small size, high mechanical strength, and long service life was selected for energy capture [5,6,7,8]. At this stage, many scholars have conducted in-depth research on piezoelectric energy capture technology using piezoelectric materials. For example, Lipeng He et al. [9] proposed a new wind energy impact energy capturer by using a new excitation method of magnetic force and external impact force to act on the cantilever beam piezoelectric oscillator, causing it to vibrate and deform, so as to capture energy. The research results showed that the device had the best performance in excitation magnet and mass magnet spacing of 5 mm, and the maximum output voltage generated was 56 V, which can be applied to the new energy field and extend the new technology of piezoelectric and magnetic coupling. Hyeong Lee et al. [10] proposed a fluid impact energy capturer, using a new way of fluid–solid coupling in the liquid for energy capturing, while using a stepped installation design can ensure a 26% reduction in stress concentration without reducing the power, the study results show that at a liquid flow rate of 20 L/min, the output power generated is 20 mW, this scheme can be applied to the field of fluid energy capturing further expands the application of piezoelectric energy capturing. Gi-Woo Kim et al. [11] proposed a fin-excited piezoelectric energy captor that uses a piezoelectric film with a Plexiglas plate to mimic an electric fish torso structure for a new type of vibration energy harvesting, and the study showed that the output power of this structural captor was 45% higher than that of a conventional vibration captor, further expanding the diversity of piezoelectric energy capturing techniques in the bionic field. Zhongjie Li et al. [12] proposed a two-degrees-of-freedom stacked piezoelectric energy capturer by using an exciter excitation amplifier to act on a new integrated piezoelectric stack, thus generating a small deformation for energy capture, and showed that when the mass was 29 g, the spring stiffness was 0.098 N/mm, and the external excitation frequency was 10.8 Hz, the resulting instantaneous output power was 521.6 mW; demonstrating this scheme extended the versatility of material forms in the field of piezoelectric energy capture. Tian-Bing Xu et al. [13] investigated the properties related to energy capture in piezoelectric stacks under resonant and non-resonant vibrations and analyzed the output voltage, output power, capacitance, impedance, and electromechanical conversion efficiency, using an external exciter acting on the piezoelectric stacks. The study showed that the piezoelectric stacks were significantly higher than the piezoelectric cantilever beam-type energy capturer of the same size, and the design scheme extended both the material versatility in the field of piezoelectric energy capture and explored the properties of the material properties. The study showed that the piezoelectric stacks are significantly higher than the piezoelectric cantilever beam-type energy capturer of the same size. However, the piezoelectric stacks are made by a laminated bonding and co-fired process and are susceptible to damage and low deformation under the reciprocating action of the exciter, resulting in low power output. Since the piezoelectric constant of piezoelectric films is about ten times that of piezoelectric oscillators, the piezoelectric films have higher electrical output characteristics compared to piezoelectric oscillators, and are more flexible during operation and have a higher service life. Piezoelectric materials can also be combined with microelectromechanical systems for energy capture and used for the self-powered needs of micro and small machines [14,15,16,17]. At present, the excitation source in piezoelectric energy-related research is relatively single and fixed, which cannot meet stable power generation under any conditions, and the environmental requirements are more stringent. Polyvinylidene fluoride has the advantages of the high sensitivity of force–electric conversion and good mechanical properties, and it can produce continuous deformation of piezoelectric films at low frequencies to generate electrical energy, which is better than piezoelectric stacks and piezoelectric oscillators for micro-vibrations [18,19,20]. The combination of energy capture using PVDF piezoelectric film aligns with the current trend of environmental protection and is green and energy-saving [21,22,23].

In this paper, we propose an impactable piezoelectric energy trap at low frequencies to trap energy by deforming a double-arch piezoelectric film through an impacting excitation force. Compared with the conventional piezoelectric stack with piezoelectric oscillator traps, the designed traps can be adapted to more applications with high-performance electromechanical conversion efficiency and deformation of the piezoelectric film during both spring compression and release. Since the designed capturer is composed of a spring, it is more sensitive to micro-vibration response and can be applied to any site with excitation force for the self-powered demand of miniature electronic devices. This study also provides a theoretical reference for subsequent work on piezoelectric energy capture.

## 2. Structural Design

### 2.1. Structure Composition

**Figure 1:**
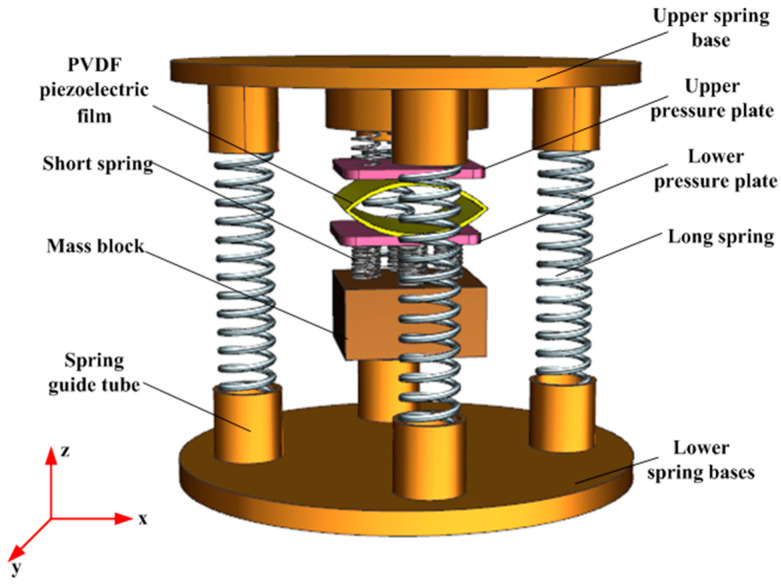
Schematic diagram of the structure of the energy capture device.

The three-dimensional structure of the impact piezoelectric energy capture device is shown in Figure 1. The piezoelectric film (polyvinylidene fluoride) adopts a double-arch structure, which can be placed by two or more upper and lower connections, and each PVDF piezoelectric film has the same structural parameters. The upper and lower pressure plates of the arch-shaped PVDF piezoelectric film are connected to each other by four short springs, and the upper and lower pressure plates and the piezoelectric film are fixed by epoxy resin adhesive. The mass block is connected to the lower pressure plate by a short spring, and the upper spring base is connected to the lower spring base by four long springs placed in the spring guide tube of the device.

**Figure 2:**
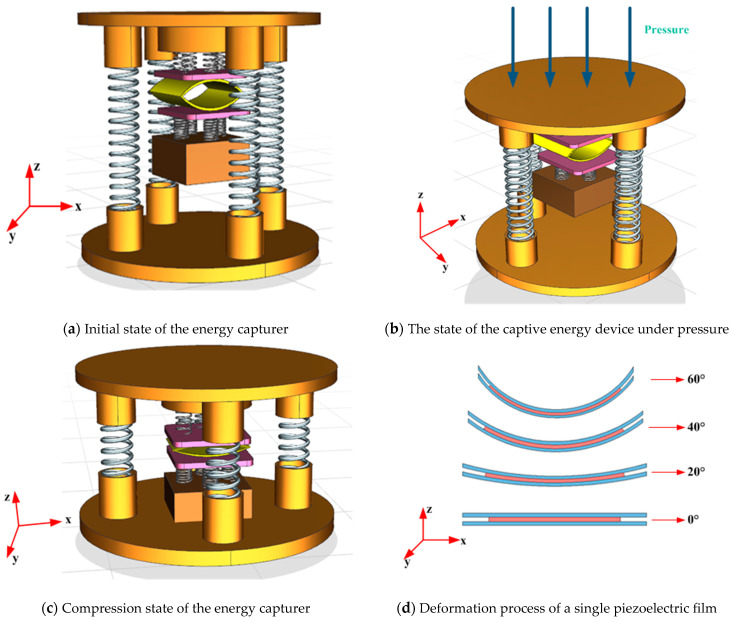
Schematic diagram of the working process of the energy capturer.

A 3D rendering of the created structural model in working condition is performed to simulate the motion of the captor under the action of external excitation. Compared with other types of energy traps, the innovation is that the excitation source is more universal and practical, and the designed piezoelectric energy trap will not be damaged by excessive external pressure but can have better power generation performance under excessive pressure. When the capturer is released under pressure, the piezoelectric film will undergo secondary stretching due to the action of the mass block to make it deformed and able to meet the energy capture under low frequency and micro-vibration response. The specific working process is shown in Figure 2.

### 2.2. Working Principle

Working principle: As shown in Figure 2b, when using external excitation force on the experimental device for impact pressure, the upper spring base will produce an axial force on the external long spring for compression, to be the mass of the experimental device and the lower spring base contact, the short spring will be compressed, so that the arch-shaped PVDF piezoelectric film deformation for piezoelectric energy capture, the collected energy for rectification, storage, and use. Since the performance of the energy capture device depends on the (polyvinylidene fluoride) piezoelectric film, the kinetic model of the energy capture device with the piezoelectric film subjected to external excitation is analyzed.

**Figure 3:**
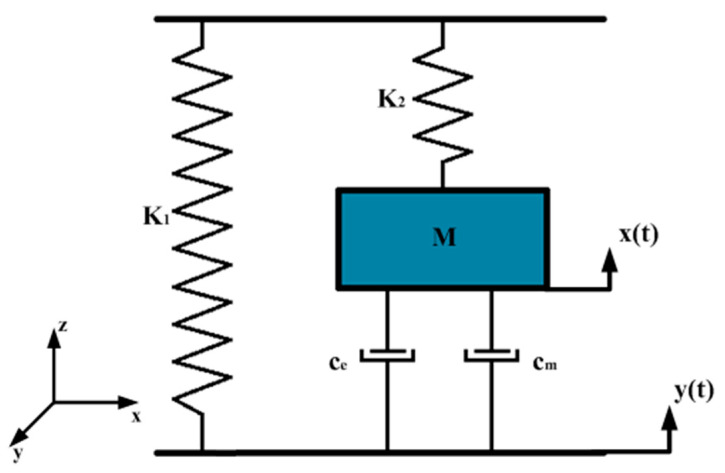
Spring-mass-damping dynamics model of the impact energy capturer.

When an impact piezoelectric energy trap is externally excited, the trap is viewed as a spring-mass-damping dynamics model, as shown in Figure 3. Where m is the mass of the mass block, the piezoelectric film, and the pressure plate, the damping is set to c, the mechanical damping between the piezoelectric film and the pressure plate is set to $c_{e}$, the system damping of the captive energy device during vibration is $c_{m}$, and the equivalent stiffness of the spring is k.

The total equivalent damping of the system is:

(1) $$c=c_{e}+c_{m}.$$

When the external excitation $y(t)=A_{0}e^{iwt}$ acts in the vibration system, the total vibration equation of the system is:

(2) $$m\ddot{x}(t)+c\dot{x}(t)+kx(t)=-m\ddot{y}(t).$$

The resulting analytical solution is given by:

(3) $$x={\bar{x}}_{0}e^{i\omega t}.$$

Combining Equations (2) and (3), the complex amplitude ${\bar{x}}_{0}$ is obtained as:

(4) $${\bar{x}}_{0}=\frac{A_{0}{\left(\frac{\omega }{\omega_{n}}\right)}^{2}}{1+2\xi \frac{\omega }{\omega_{n}}-{\left(\frac{\omega }{\omega_{n}}\right)}^{2}}.$$

The amplitude of vibration of the mass block m is:

(5) $${\bar{x}}_{0}=\frac{A_{0}{\left(\frac{\omega }{\omega_{n}}\right)}^{2}}{\sqrt{{\left[1-(\frac{\omega }{\omega_{n}})\right]}^{2}-{\left(2\xi \frac{\omega }{\omega_{n}}\right)}^{2}}}=\frac{A_{0}\lambda^{2}}{\sqrt{{\left[1-\lambda \right]}^{2}+{\left(2\xi \lambda \right)}^{2}}}$$

where $A_{0}$ is the source amplitude, ω is the external excitation frequency, $\omega_{n}$ is the intrinsic frequency of the system, ξ is the system damping ratio, $\lambda =\frac{\omega }{\omega_{n}}$, $\xi =\frac{c}{2mw_{n}}$, $\omega_{n}=\sqrt{\frac{k_{1}+k_{2}}{m}}$.

The total damping force to be overcome for stable operation of the system is:

(6) $$F_{c}=\left(c_{e}+c_{m}\right)\dot{x}(t).$$

In one cycle, the work done by the energy capturer to overcome the resistance is:

(7) $$w=\int_{0}^{\frac{2\pi }{\omega }}F_{c}dx(t)=\int_{0}^{\frac{2\pi }{\omega }}\left(c_{e}+c_{m}\right)\dot{x}(t)dx(t).$$

Substituting Equation (3) into Equation (7), we obtain:

(8) $$w=\left(c_{e}+c_{m}\right)x_{0}{}^{2}\pi \omega$$

The power of the system can be obtained as:

(9) $$P=\frac{w}{T}=\frac{m\left(\xi_{e}+\xi_{m}\right)\omega^{3}A_{0}{}^{2}\lambda^{3}}{{\left(1-\lambda^{2}\right)}^{2}+{\left[2\left(\xi_{e}+\xi_{m}\right)\lambda \right]}^{2}}.$$

**Figure 4:**
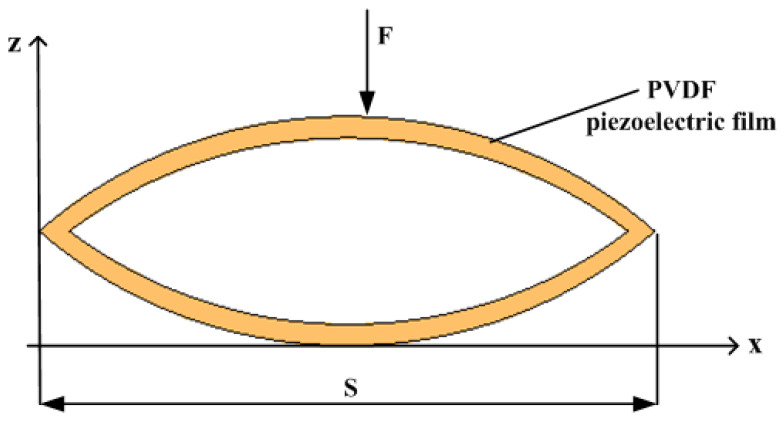
Schematic diagram of PVDF piezoelectric film structure.

Parametric modeling of the piezoelectric film is performed, and the mechanical model of its piezoelectric film is shown in Figure 4. Where $h_{f}$ is the thickness of PVDF piezoelectric film, $h_{t}$ is the thickness of material PET, s is the length of piezoelectric film, W is the width of piezoelectric film, h is the height of piezoelectric film, F is the external excitation force, and $\partial =h_{f}/ht$ is the thickness ratio of the piezoelectric film.

When a PVDF piezoelectric film is subjected to an external excitation F, the piezoelectric film is deformed by a downward force, and the piezoelectric equation is:

(10) $$\sigma_{x}=E_{p}\left(\delta_{1}-d_{31}B\right)$$

(11) $$E=-d_{31}\sigma_{x}+\beta_{33}{}^{T}B$$

where $\sigma_{x}$ is the stress in the X-direction, $E_{p}$ is the Young’s modulus of the PVDF piezoelectric film, $\delta_{1}$ is the strain in the x-direction ($\delta_{1}=Sz$, S is the radius of curvature), B is the potential shift of the piezoelectric film in the Z-direction, $d_{31}$ is the piezoelectric constant, E is the electric field strength of the piezoelectric film, and $\beta_{33}{}^{T}$ is the dielectric isolation rate.

The radius of curvature of the piezoelectric film is:

(12) $$S=-\frac{3}{GEpwh^{3}\lambda_{z}}\left[4(x-s)F+(1-\partial^{2})Wh^{2}d_{31}EpB\right]$$

where $G=1-\partial^{3}+\partial^{3}\beta$, $\beta =E_{t}/E_{p}$, $\lambda_{z}=\frac{s}{2}\tan\theta$, $E_{t}$ are the Young’s modulus of PET, and $\lambda_{z}$ is the bending deflection of the piezoelectric film.

Integrating the obtained electric field strength in the Z-direction, the voltage is obtained as:

(13) $$V=f\int_{\frac{h}{2}-h_{t}}^{\frac{h}{2}}Edz=\frac{f(1-\partial )}{8GWh\lambda_{z}}\left[12(1+\partial )\;d_{31}(s-x)F+RWh^{2}\beta_{33}^{T}B\right]$$

Among them:

(14) $$R=-3(1-\partial ){\left(1+\partial \right)}^{2}k_{33}^{2}+4G(1+k_{33}^{2})$$

Changing the voltage in (13) to the equation for potential shift:

(15) $$B=\frac{8GWhV-12f(1-\alpha^{2})d_{31}(s-x)F\lambda_{z}}{(1-\partial )RWh^{2}\beta_{33}^{T}}$$

Integration of the potential shift yields the charge as:

(16) $$Q=2\int_{0}^{W}\int_{0}^{s}Bdydx=\frac{16GsWV}{\beta_{33}^{T}Rh(1-\alpha )}-\frac{12s^{2}f(1+\partial )d_{31}F\lambda_{z}}{\beta_{33}^{T}Rh^{2}}$$

The above equation is calculated under the simultaneous action of external force and self-excited electric field, while in general, the external force under the action of the piezoelectric device can ignore the internal electric field. Therefore, the amount of charge generated can be expressed as:

(17) $$Q=-\frac{12s^{2}(1+\partial )d_{31}F\lambda_{z}}{\beta_{33}^{T}Rh^{2}}$$

The free capacitance of the piezoelectric film in the self-excited electric field can be obtained from Equation Q=CV and Equation (16) as:

(18) $$C=\frac{16GWs}{(1-\partial )\beta_{33}^{T}Rh}$$

Thus the output voltage of a single piezoelectric film is:

(19) $$V=-\frac{3f(1-\partial^{2})d_{31}Fs\lambda_{z}}{4GWh}$$

In order to optimize the structure and volume of the piezoelectric film, the power density is, therefore, used as an important parameter for the energy harvesting of the piezoelectric cell, which can be expressed as:

(20) $$\phi =\frac{P}{V^{*}}$$

where ϕ is the power density, P is the film output power, and $V^{*}$ is the volume of the piezoelectric film.

According to equation P=UI, by substituting Equation (19) into Equation (20), the power density ϕ can be further obtained as:

(21) $$\phi =\frac{-3f(1-\partial^{2})d_{31}F\lambda_{z}I}{4Gw^{2}h^{2}}$$

From Equations (19) and (21), it can be seen that the power generation of piezoelectric films is positively related to the bending amplitude in the power density and is affected by the dimensional parameters, external excitation, and material properties.

**Figure 5:**
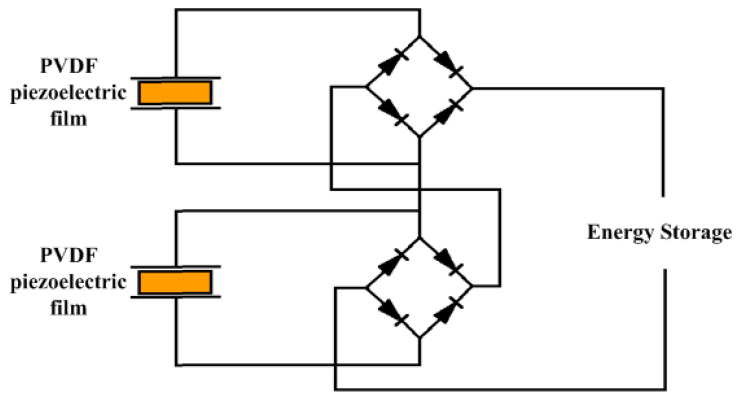
Series rectifier circuit diagram.

In order to make the power generation module with higher power generation performance, the power generation module in this experimental setup consists of two arch-shaped PVDF piezoelectric films assembled in series. When PVDF piezoelectric film is subjected to the action of external force F, the voltage and current of its piezoelectric film have alternating characteristics, and we need a regulated power supply for charging the electrical appliances around us in daily life, so we have to rectify it. The rectification circuit will avoid the phenomenon that the PVDF piezoelectric film of high voltage charges the PVDF piezoelectric film of low voltage and its circuit diagram when the PVDF piezoelectric film is connected in series, as shown in Figure 5.

Since the prototype designed for the impact piezoelectric trap consists of several springs connected, the stiffness of the springs needs to be analyzed in order to ensure that the piezoelectric film can be subjected to sufficient impact forces. Let the initial height of the spring be $h_{1}$, the height of the spring after installation be $h_{2}$, the compression of the spring when the mass is falling freely be $L_{1}=h_{1}-h_{2}+\gamma_{1}$, and the compression of the spring when the mass is in contact with the lower spring substrate be $L_{2}=h_{1}-h_{2}+\gamma_{1}-\gamma_{2}$. Therefore the mechanical energy of the mass block before collision with the spring substrate is:

(22) $$T=\frac{1}{2}k_{2}L^{2}{}_{1}-\frac{1}{2}k_{2}L^{2}{}_{2}+mg\gamma_{2}$$

Without considering the energy loss, the law of conservation of mechanical energy yields:

(23) $$\frac{1}{2}k_{2}L^{2}{}_{1}-\frac{1}{2}k_{2}L^{2}{}_{2}+mg\gamma_{2}=\frac{1}{2}F_{D}\Delta_{1}$$

where $F_{D}$ is the captive energy impact load and $\Delta_{1}=\frac{F_{D}}{k_{D}}$ is the mechanism deformation under the impact load.

From Hooke’s law, we can obtain:

(24) $$F_{D}=kF_{J}$$

(25) $$\Delta_{D}=k\Delta_{J}$$

where k is the dynamic load factor, $F_{J}=k_{2}L_{2}+mg$, and $\Delta_{J}=\frac{F_{J}}{k_{D}}$ are the institutional deformation variables of the model under static action.

According to Equations (23) and (25) we can obtain:

(26) $$K=\sqrt{\frac{k_{D}(k_{2}L^{2}{}_{1}-k_{2}L^{2}{}_{2}+2mg\gamma_{2})}{{\left(k_{2}L_{2}+mg\right)}^{2}}}.$$

Therefore the spring should meet $KF_{J}\leq F_{D}{}_{\max}$, where $F_{D}{}_{\max}$ is the maximum impact load that the captive can withstand.

## 3. Simulation and Theoretical Analysis

In order to verify the theoretical soundness of the designed energy trap, the energy trap was parametrically modeled by using UG 3D software, and the power generation performance of the energy trap and piezoelectric module was simulated and analyzed by using COMSOL Multiphysics simulation software. During the simulation analysis, the influence of the adhesion layer on the power generation performance of the piezoelectric material was ignored, the material of the spring substrate and pressure plate was selected as the sub-glide plate, and the material of the mass block and spring was selected as alloy steel. The material properties of its impact energy capturer are shown in Table 1.

### 3.1. Modal Analysis of Energy Capturer

**Figure 6:**
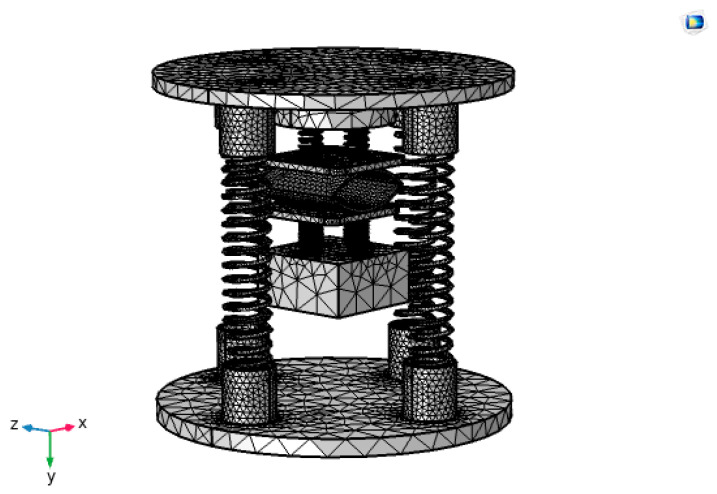
Meshing of PVDF piezoelectric film.

**Figure 7:**
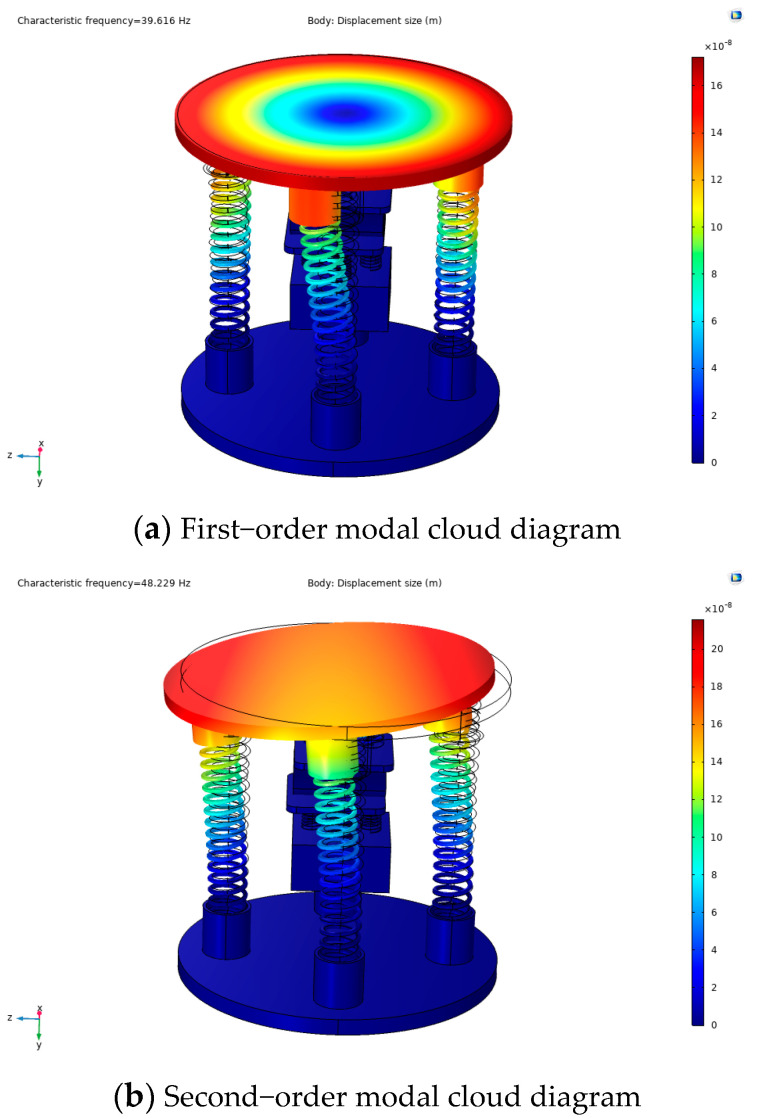

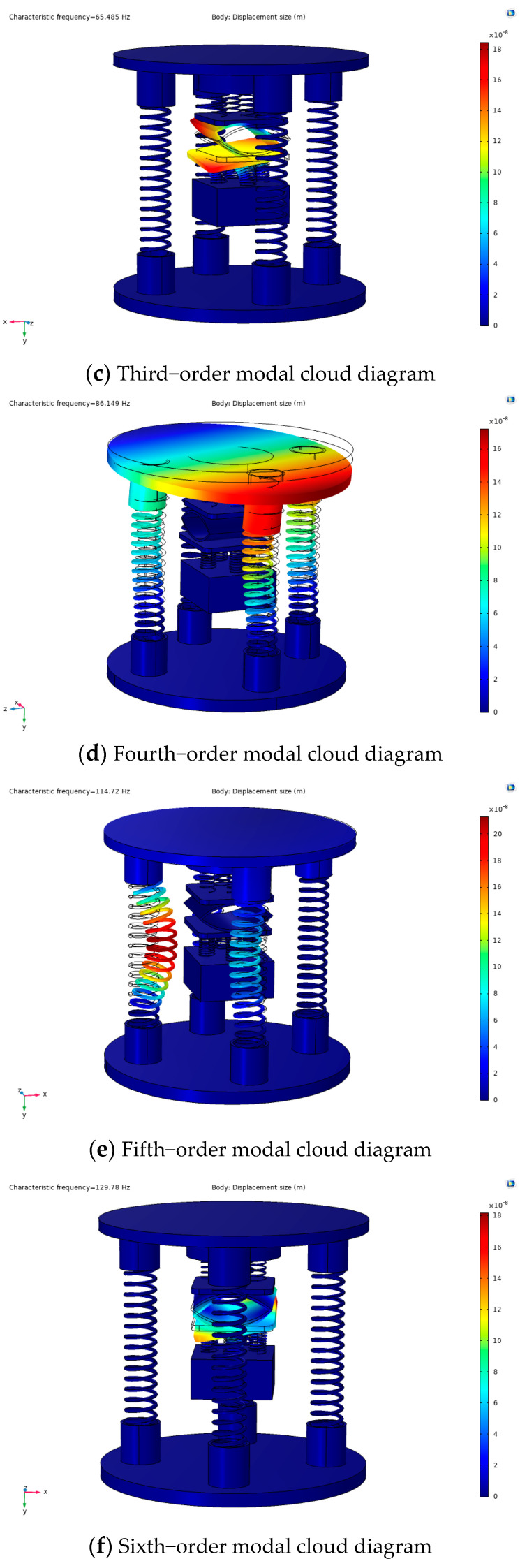
Cloud diagram of the first sixth-order mode of the energy trap.

Since this experimental study is on energy harvesting under non-resonance, the frequency characteristics under resonance need to be simulated through simulation to provide theoretical support for subsequent non-resonance experiments. According to the kinetic model, the capturer mainly consists of a spring and a mass block. When the capturer is externally excited, the piezoelectric module is forced to vibrate with the spring. The spring-mass-damping system enables us to obtain the differential equation of the vibration of the captive energy device and, thus, the input–output response of the captive energy device under external excitation. Therefore, to know the actual operating state of the captive energy, it is necessary to perform a modal analysis of its captive energy. Its modal analysis is also the starting point for the study of forced vibration devices by transient analysis and harmonic response analysis. Therefore, the COMSOL Multiphysics simulation software was used to fix constraints on the lower spring substrate in the physical field, and all springs were set as spring bases in the physical field, and the overall free tetrahedral mesh was used for the partitioning, as shown in Figure 6. Since the frequency generated by the capturer itself is not too high during operation, only the modal analysis of the first six orders is considered, and the simulation results are shown in Figure 7.

As can be seen in Figure 7, the first six orders of intrinsic frequencies of the energy capturer were 39.616 Hz, 48.229 Hz, 65.485 Hz, 86.149 Hz, 114.72 Hz, and 129.78 Hz, respectively. At the first-order mode of the capturer, the capturer had the lowest intrinsic frequency, which is more consistent with the deformation when it is excited. Although the designed capsule is used in various environments where excitation forces are present, in most cases, the frequency does not reach the lowest value of its intrinsic frequency, but in special places, it can reach its intrinsic frequency. Its modal analysis is also the starting point for the harmonic response analysis of forced vibration calculation, so the simulation analysis of the first six orders of the modal of the energy capturer is of great significance for the future study of vibration energy harvesting.

### 3.2. Stress–Strain Analysis of Energy Traps

**Figure 8:**
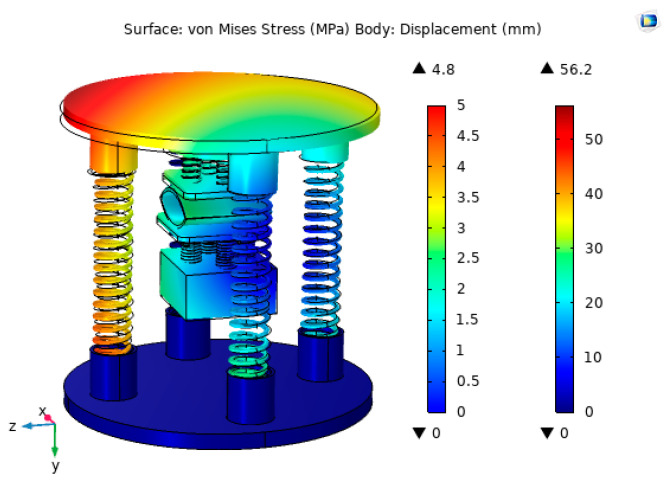
Stress−strain cloud of the energy trap.

The structural strength of the capsule is analyzed using COMSOL Multiphysics simulation software in order to ensure the proper operation of the capsule due to the continuous external excitation during operation. First, the bottom surface of the lower spring substrate of the energy trap was fixedly restrained. Next, a body load of 30 N was applied to the upper spring substrate by choosing a body load in the physical field, which was divided using a free tetrahedral mesh. Finally, the study was solved for the steady state. The simulation results are shown in Figure 8.

From Figure 8, it can be seen that the stress generated by the captive energy device under the applied body load of 30 N was 4.8 MPa and the output displacement was 56.2 mm. The resulting stress was much less than the permissible stress of the sub-grid plate (77 Mpa), which meets the experimental requirements.

### 3.3. Voltage Simulation Analysis of the Piezoelectric Modules

**Figure 9:**
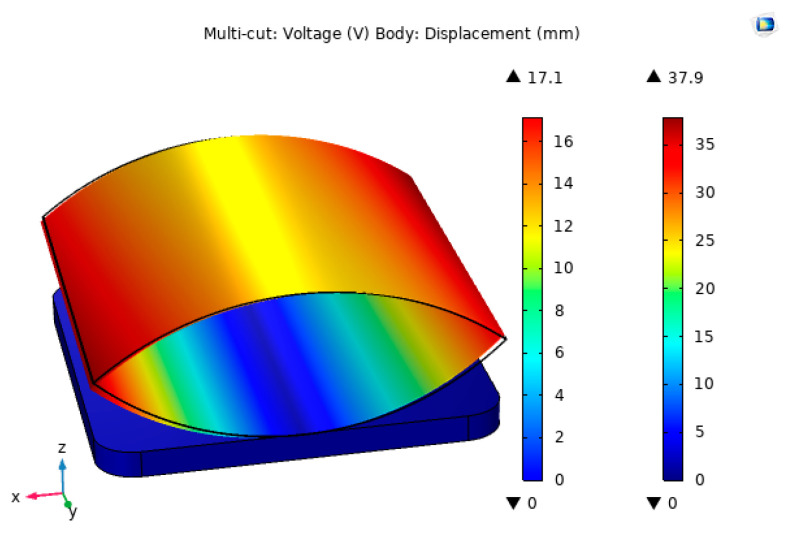
Voltage diagram of the experimental setup.

The piezoelectric film size of 20 × 40 mm was selected, and the piezoelectric module in the impact trap was simulated and analyzed for output voltage using COMSOL Multiphysics simulation software. Firstly, the contact surface between the lower pressure plate and the piezoelectric film was fixedly constrained, and the lower surface of the piezoelectric film was grounded in the electrostatic field, and secondly, a boundary load of 30 N was applied to the upper surface of the piezoelectric film, which was divided using a free tetrahedral mesh. Finally, the steady-state solution of the piezoelectric module was performed. The simulation results of the generated output voltage are shown in Figure 9.

As can be seen from Figure 9, the maximum output voltage generated by the piezoelectric module was 17.1 V and the output displacement generated under a 30 N boundary load was 37.9 mm. The output voltage obtained from the simulation was more ideal and can meet the experimental requirements.

**Figure 10:**
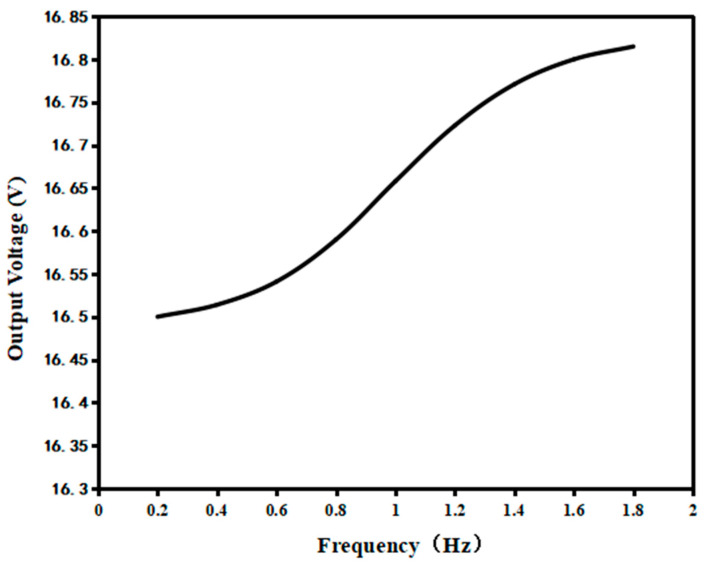
The characteristic curve of the output voltage at different frequencies.

In order to be closer to the real operation of the trap, when two PVDF piezoelectric films were connected in series, the excitation frequency was set to 0.2–2 Hz because the external excitation number set by the impact piezoelectric trap through the signal generator was about 30–40 times per minute. Thus, the voltage output characteristics of the energy capturer at different frequencies were further investigated, as shown in Figure 10.

As can be seen from Figure 10, in the frequency range of 0.2–2 Hz, the faster the frequency of the excitation force, the higher the output voltage, but the difference was not too obvious. The maximum value of the output voltage was only 0.319 V different from the minimum value. The above situation may occur because of the increase in excitation frequency, while the impact force remains the same, causing a slight difference in the state of the piezoelectric unit when deformation and recovery are performed. However, as the frequency increased, the deformation of the reciprocating piezoelectric module increased, resulting in a slight increase in the output voltage generated by the capturer, so the increase is not very significant.

## 4. Experimental Testing

### 4.1. Experimental Setup and Test Method

**Figure 11:**
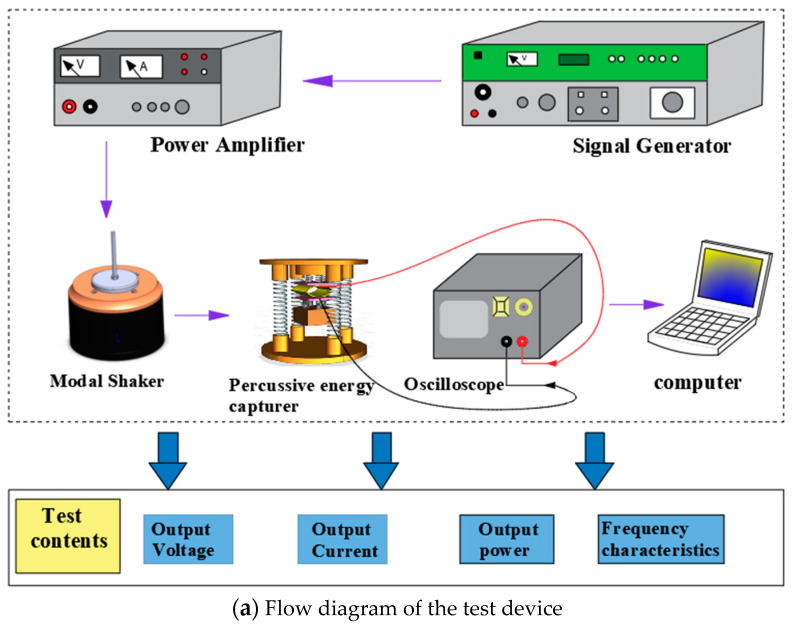

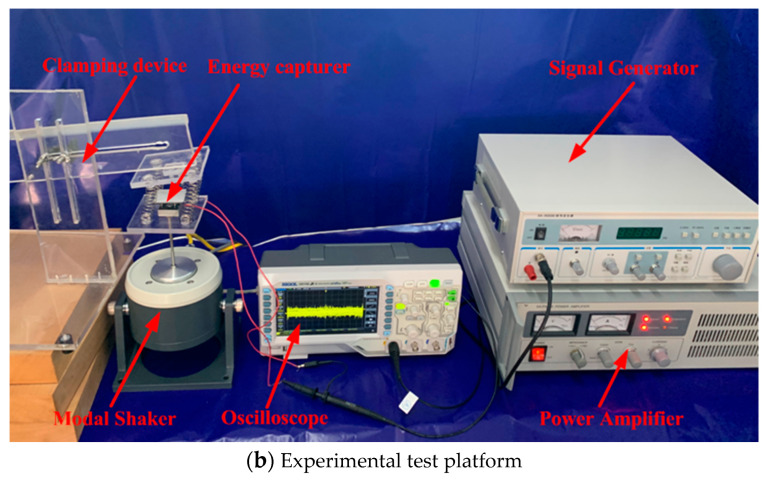
Schematic diagram of the impact capturer test.

In order to verify the feasibility of the theoretical energy capturer model, the experimental prototype was machined using a precision engraving machine and the experimental platform was built. The flow diagram of the test setup is shown in Figure 11a, and the experimental test platform of the impact piezoelectric energy capturer is shown in Figure 11b. It mainly includes a capturer prototype, clamping device, modal exciter (SA-JZ005T), signal generator (SA-SG030), power amplifier (SA-PA010), oscilloscope (RIGOL-DS1102), and computer. Experimental method: After the experimental prototype is assembled and fixed with the clamping device, start the power supply of the oscilloscope, power amplifier, and signal generator, connect the piezoelectric film with the oscilloscope, adjust the signal generator and power amplifier to make the exciter apply the excitation force at the bottom of the lower spring base, record the peak data generated by the energy capturer in the oscilloscope, and transfer the data sampling to the computer for processing. The characteristic relationship curves of output voltage, output current, and output power generated by different bending amplitude and film size in low-frequency environments are tested by oscilloscope.

### 4.2. Test of Bending Amplitude on Output Voltage

**Figure 12:**
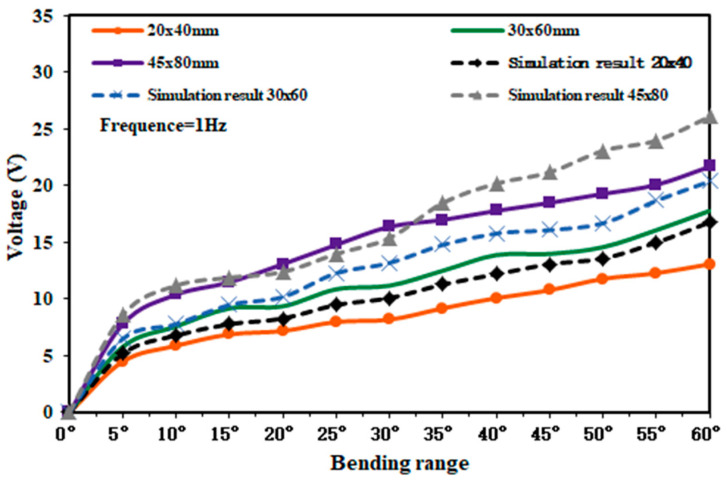
Output voltage characteristic curve of three sets of piezoelectric films.

Since the bending amplitude and size of the piezoelectric film in the capturer have a great influence on the output voltage. Therefore, the selected piezoelectric film sizes are 20 × 40 mm, 30 × 60 mm, and 45 × 80 mm. By changing the bending amplitude of the piezoelectric film, the magnitude of the output voltage under different film angles was measured by using an oscilloscope when the external excitation force was 30 N. The experimental test and simulation results are shown in Figure 12.

As can be seen from Figure 12, when the PVDF piezoelectric film was bent at 60° with respect to the plane and the size was 45 × 80 mm, the peak output voltage generated by the capturer was 21.69 V, and it had some stability in this range. The reasonableness of the simulation model is verified by forming a comparison with the simulation results.

### 4.3. Test of Bending Amplitude on Output Current

**Figure 13:**
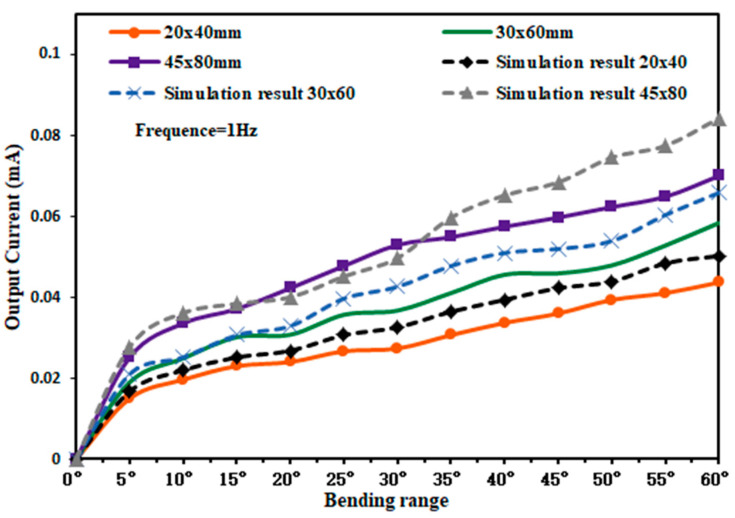
Output current characteristic curve of three sets of piezoelectric films.

The piezoelectric film sizes of 20 × 40 mm, 30 × 60 mm, and 45 × 80 mm were selected and measured by using the above experimental method, changing the bending amplitude of the piezoelectric film and the horizontal surface, and measuring the output current of different size capturers by using the probe of the oscilloscope. The experimental test and simulation results are shown in Figure 13.

As can be seen from Figure 13, the output current generated by the three sizes of piezoelectric films tends to rise as the bending amplitude gradually increases. When the piezoelectric film was bent at 60°, the output current is 0.0433 mA for a 20 × 40 mm piezoelectric film, 0.0553 mA for a 30 × 60 mm piezoelectric film, and 0.07 mA for a 45 × 80 mm piezoelectric film. The maximum output currents of the piezoelectric films obtained from the simulation were 0.0502 mA, 0.0658 mA, and 0.0842 mA, respectively.

### 4.4. Test of Bending Amplitude on Output Power

**Figure 14:**
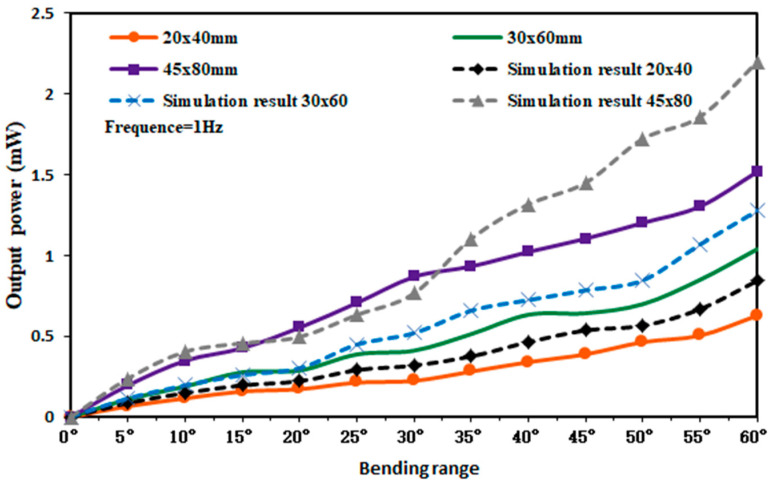
Output power characteristic curves of three groups of piezoelectric films.

From Figure 12 and Figure 13, the output voltage and output current of the capturer during the operation can be obtained. According to equation P=UI, the characteristic curve of the output power of the capturer can be obtained, and the experimental test and simulation results are shown in Figure 14.

As can be seen from Figure 14, when the bending amplitude of the piezoelectric film in the capturer was 60°, the output power of the 20 × 40 mm piezoelectric film capturer was 0.6276 mW, the output power of the 30 × 60 mm piezoelectric film capturer was 1.0395 mW, and the output power of the 45 × 80 mm piezoelectric film capturer was 1.5176 mW. The maximum output powers of the piezoelectric films obtained from the simulation were 0.844 mW, 1.277 mW, and 2.197 mW, respectively. The obtained simulation results were higher than the experimental results in the test, on the one hand, may be due to the fact that the output voltage and output current obtained from the simulation were under the ideal environment, ignoring the internal variation of the piezoelectric layer and the adhesion layer, making the layered structure non-conducting, thus producing this phenomenon. On the other hand, the piezoelectric film was deformed by the external force, which leads to losses in the process of charge generation with the connected circuit, and, thus, the experimental results are lower than the simulation results.

### 4.5. Effect of Different Excitation Forces on Output Voltage

**Figure 15:**
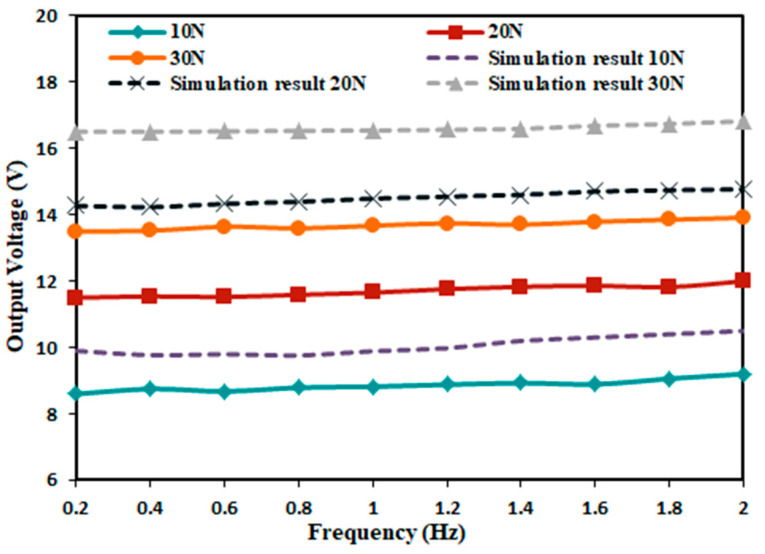
Output voltage characteristic curve under different excitation force.

Since the designed impact piezoelectric energy capturer can be applied in different environments, the excitation force generated in different environments is different. Therefore, the above experimental setup was used to test the performance of the output voltage produced by the energy capturer under different excitation forces. In order to ensure that the capturer can work properly and facilitate the measurement, the piezoelectric film size of 20 × 40 mm, the excitation force of 10 N, 20 N, 30 N, the film bending amplitude of 60°, and the excitation frequency of 0.2–2 Hz were selected for the test, and the peak voltage at each set of frequency was recorded and compared with the simulation results. The results of the experimental tests are shown in Figure 15.

As can be seen from Figure 15, the output voltages produced by the energy capturer are different at different excitation forces, and the output voltage increases gradually as the excitation force increases, but the increase gradually decreases. The three excitation forces gradually increase the output voltage with the increase of driving frequency, but the increase is not too obvious, and the difference between the maximum value and the minimum value is 0.6 V. The reasonableness of the simulation model is further verified by comparing it with the simulation results.

### 4.6. Comparative Testing of Energy Capture Devices

**Figure 16:**
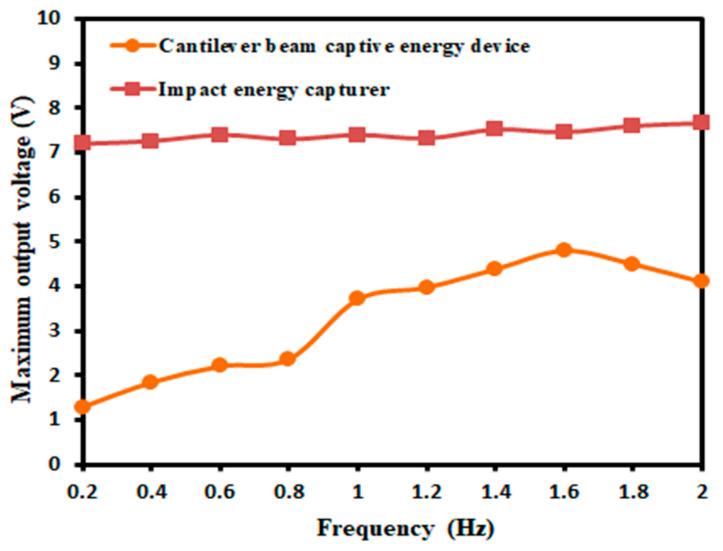
Comparison test of energy traps at different excitation frequencies.

In order to verify the better output performance of the designed device than the conventional energy capturer at low frequencies, the conventional cantilever beam-type piezoelectric oscillator capturer was selected for comparison tests. The 50 × 15 × 0.3 ${\mathrm{mm}}^{3}$ piezoelectric oscillator was first fixed to the bracket and the excitation frequency was set to 0.2–2 Hz using a signal generator and transmitted to a power amplifier. The amplitude of the exciter was adjusted by the signal generator to ensure the normal operation of the piezoelectric oscillator so that the two types of captors are subjected to the same degree of deformation, and the maximum output voltage generated by the two types of captors at different excitation frequencies was tested. The experimental results are shown in Figure 16.

From Figure 16, it can be seen that the designed impact energy capturer produces a higher output voltage than the cantilever beam piezoelectric energy capturer, and the variation range had a certain stability. In the application of piezoelectric oscillators, they are prone to brittle fracture and are not suitable for applications with high excitation force, while the designed piezoelectric film had the characteristics of good flexibility and high service life, which can be applied in most environments.

## 5. Conclusions

This paper proposes an impact piezoelectric energy capture device, mainly composed of a piezoelectric film, spring, spring base, mass block, pressure plate, where the spring-type structure can be better applied to the presence of an excitation force, while the flexibility of the piezoelectric film can better ensure the service life of the device and improve the electromechanical conversion efficiency. In this experiment, the working principle and structural composition of the energy trap are first analyzed, and the modal, stress–strain, and output voltage of its trap are simulated and analyzed using COMSOL Multiphysics software to obtain the deformation of the piezoelectric trap during operation. The prototype is then machined and fabricated using a precision engraving machine, and finally, the relevant experimental tests are performed. The experimental results show that the output power voltage is 21.69 V, the output current is 0.07 mA, and the output power is 1.5176 mW when the external excitation force is 30 N, the bending amplitude is 60°, and the size of the piezoelectric film is 45 × 80 mm. This experiment verifies the feasibility of an impact piezoelectric energy captor that can be placed in an environment with external shocks, has the property of generating energy, and provides a new idea for powering electronic components.

## Figures and Tables

**Table 1:** Material properties of energy traps.

Materials	Properties	Numerical Value
PVDF	Density (kg/m³)	1780
	Modulus of elasticity (GPa)	2.8
	Poisson’s ratio	0.33
Acrylic sheet	Density (kg/m³)	1190
	Young’s modulus (GPa)	3.2
	Poisson’s ratio	0.35
	Coefficient of thermal expansion W/(m·K)	0.18
Alloy Steel	Density (kg/m³)	7850
	Young’s modulus (GPa)	200
	Poisson’s ratio	0.3
	Relative dielectric constant	1
	Thermal conductivity W/(m·K)	44.5

## Data Availability

The data that support the findings of this study are available from the corresponding author upon reasonable request.
